# Supplementary material for: Perifascial areolar tissue graft promotes angiogenesis and wound healing in an exposed ischemic component rabbit model
Source: PLoS One. 2024 Feb 20;19(2):e0298971. doi: 10.1371/journal.pone.0298971 (PMC10878522; doi:10.1371/journal.pone.0298971)
Supplement: Attachment — Submitted filename: Response to the comments of the Reviewer.docx [file pone.0298971.s003.docx]

**Response to the comments of the Reviewer**

Dear Editor,

Thank you for evaluating our manuscript, your appreciation of our work,you’re your encouraging comments. We appreciate your comments and the reviewers’ guidance and thoughtful suggestions from which the manuscript has benefited. We have read the comments provided by you and the reviewers and have revised the manuscript accordingly. Please find our point-by-point responses to all comments below.

**Editorial remarks:**

1. Please ensure that your manuscript meets PLOS ONE's style requirements, including those for file naming. The PLOS ONE style templates can be found at https://journals.plos.org/plosone/s/file?id=wjVg/PLOSOne_formatting_sample_main_body.pdf and <https://journals.plos.org/plosone/s/file?id=ba62/PLOSOne_formatting_sample_title_authors_affiliations.pdf>

(Response)

We have adhered to the specified style requirements. Should you identify any areas for correction or improvement, please do not hesitate to inform us.

2. Thank you for stating the following financial disclosure: "This research received support from the Japan Society for the Promotion of Science (JSPS) KAKENHI Grant Number 19K12809 and an SBC Research Support Grant. "Please state what role the funders took in the study. If the funders had no role, please state: "The funders had no role in study design, data collection and analysis, decision to publish, or preparation of the manuscript." If this statement is not correct you must amend it as needed. Please include this amended Role of Funder statement in your cover letter; we will change the online submission form on your behalf.

(Response)

We have included this information in the cover letter accordingly.

3. In your Data Availability statement, you have not specified where the minimal data set underlying the results described in your manuscript can be found. PLOS defines a study's minimal data set as the underlying data used to reach the conclusions drawn in the manuscript and any additional data required to replicate the reported study findings in their entirety. All PLOS journals require that the minimal data set be made fully available. For more information about our data policy, please see <http://journals.plos.org/plosone/s/data-availability>.

(Response)

Minimal data sets have been updated in the Supporting Information file accordingly.

4. Please amend either the title on the online submission form (via Edit Submission) or the title in the manuscript so that they are identical.CMBL-D-18-00111
   Angiogenesis after administration of basic fibroblast growth factor induces proliferation and differentiation of mesenchymal stem cells in elastic perichondrium in an in vivo model: Review of three sequential republication-abridged reports
   Toru Miyanaga, M.D., Ph.D; Yoshimichi Ueda; Aiko Miyanaga; Mikio Yagishita; Naoko Hama
   Cellular & Molecular Biology Letters

   Dear Dr. Miyanaga,

   Your manuscript "Angiogenesis after administration of basic fibroblast growth factor induces proliferation and differentiation of mesenchymal stem cells in elastic perichondrium in an in vivo model: Review of three sequential republication-abridged reports" (CMBL-D-18-00111) has been assessed by our reviewers.

   I am glad to inform you that the manuscript could become acceptable for publication provided the authors would address all points raised by referees in details. Their reports, together with Editorial comments, are below.

   If you are able to fully address these points, we would encourage you to submit a revised manuscript to Cellular & Molecular Biology Letters. Once you have made the necessary corrections, please submit online at:

   https://cmbl.editorialmanager.com/

   If you have forgotten your username or password please use the "Send Login Details" link to get your login information. For security reasons, your password will be reset.

   Please include a point-by-point response within the 'Response to Reviewers' box in the submission system and highlight (with 'tracked changes'/coloured/underlines/highlighted text) all changes made when revising the manuscript. Please ensure you describe additional experiments that were carried out and include a detailed rebuttal of any criticisms or requested revisions that you disagreed with. Please also ensure that your revised manuscript conforms to the journal style, which can be found in the Submission Guidelines on the journal homepage.

   The due date for submitting the revised version of your article is 30 Aug 2018.

   Please note, if your manuscript is accepted you will not be able to make any changes to the authors, or order of authors, of your manuscript once the editor has accepted your manuscript for publication. If you wish to make any changes to authorship before you resubmit your revisions, please reply to this email and ask for a 'Request for change in authorship' form which should be completed by all authors (including those to be removed) and returned to this email address. Please ensure that any changes in authorship fulfil the criteria for authorship as outlined in BioMed Central's editorial policies (http://www.biomedcentral.com/about/editorialpolicies#authorship).

   Once you have completed and returned the form, your request will be considered and you will be advised whether the requested changes will be allowed.
   By resubmitting your manuscript you confirm that all author details on the revised version are correct, that all authors have agreed to authorship and order of authorship for this manuscript and that all authors have the appropriate permissions and rights to the reported data.

   Please be aware that we may investigate, or ask your institute to investigate, any unauthorised attempts to change authorship or discrepancies in authorship between the submitted and revised versions of your manuscript.

   I look forward to receiving your revised manuscript soon.

   Best wishes,
   Aleksander F. Sikorski, Professor
   Cellular & Molecular Biology Letters
   https://cmbl.biomedcentral.com/

   Editorial remarks:

   1. The manuscript could become acceptable for publication only as Mini review. So please change the type of manuscript from Review to Mini review during resubmission.

   2. In Figs 5 and 6 please insert large A and B.

   3. Please remove "Fig. 6A" and "Fig. 6B" and type only "A" and "B". The legend should be: Common title. A...., B....

   4. In legends, where necessary, please add "With permission ...." and please add full reference.CMBL-D-18-00111
   Angiogenesis after administration of basic fibroblast growth factor induces proliferation and differentiation of mesenchymal stem cells in elastic perichondrium in an in vivo model: Review of three sequential republication-abridged reports
   Toru Miyanaga, M.D., Ph.D; Yoshimichi Ueda; Aiko Miyanaga; Mikio Yagishita; Naoko Hama
   Cellular & Molecular Biology Letters

   Dear Dr. Miyanaga,

   Your manuscript "Angiogenesis after administration of basic fibroblast growth factor induces proliferation and differentiation of mesenchymal stem cells in elastic perichondrium in an in vivo model: Review of three sequential republication-abridged reports" (CMBL-D-18-00111) has been assessed by our reviewers.

   I am glad to inform you that the manuscript could become acceptable for publication provided the authors would address all points raised by referees in details. Their reports, together with Editorial comments, are below.

   If you are able to fully address these points, we would encourage you to submit a revised manuscript to Cellular & Molecular Biology Letters. Once you have made the necessary corrections, please submit online at:

   https://cmbl.editorialmanager.com/

   If you have forgotten your username or password please use the "Send Login Details" link to get your login information. For security reasons, your password will be reset.

   Please include a point-by-point response within the 'Response to Reviewers' box in the submission system and highlight (with 'tracked changes'/coloured/underlines/highlighted text) all changes made when revising the manuscript. Please ensure you describe additional experiments that were carried out and include a detailed rebuttal of any criticisms or requested revisions that you disagreed with. Please also ensure that your revised manuscript conforms to the journal style, which can be found in the Submission Guidelines on the journal homepage.

   The due date for submitting the revised version of your article is 30 Aug 2018.

   Please note, if your manuscript is accepted you will not be able to make any changes to the authors, or order of authors, of your manuscript once the editor has accepted your manuscript for publication. If you wish to make any changes to authorship before you resubmit your revisions, please reply to this email and ask for a 'Request for change in authorship' form which should be completed by all authors (including those to be removed) and returned to this email address. Please ensure that any changes in authorship fulfil the criteria for authorship as outlined in BioMed Central's editorial policies (http://www.biomedcentral.com/about/editorialpolicies#authorship).

   Once you have completed and returned the form, your request will be considered and you will be advised whether the requested changes will be allowed.
   By resubmitting your manuscript you confirm that all author details on the revised version are correct, that all authors have agreed to authorship and order of authorship for this manuscript and that all authors have the appropriate permissions and rights to the reported data.

   Please be aware that we may investigate, or ask your institute to investigate, any unauthorised attempts to change authorship or discrepancies in authorship between the submitted and revised versions of your manuscript.

   I look forward to receiving your revised manuscript soon.

   Best wishes,
   Aleksander F. Sikorski, Professor
   Cellular & Molecular Biology Letters
   https://cmbl.biomedcentral.com/

   Editorial remarks:

   1. The manuscript could become acceptable for publication only as Mini review. So please change the type of manuscript from Review to Mini review during resubmission.

   2. In Figs 5 and 6 please insert large A and B.

   3. Please remove "Fig. 6A" and "Fig. 6B" and type only "A" and "B". The legend should be: Common title. A...., B....

   4. In legends, where necessary, please add "With permission ...." and please add full reference.CMBL-D-18-00111
   Angiogenesis after administration of basic fibroblast growth factor induces proliferation and differentiation of mesenchymal stem cells in elastic perichondrium in an in vivo model: Review of three sequential republication-abridged reports
   Toru Miyanaga, M.D., Ph.D; Yoshimichi Ueda; Aiko Miyanaga; Mikio Yagishita; Naoko Hama
   Cellular & Molecular Biology Letters

   Dear Dr. Miyanaga,

   Your manuscript "Angiogenesis after administration of basic fibroblast growth factor induces proliferation and differentiation of mesenchymal stem cells in elastic perichondrium in an in vivo model: Review of three sequential republication-abridged reports" (CMBL-D-18-00111) has been assessed by our reviewers.

   I am glad to inform you that the manuscript could become acceptable for publication provided the authors would address all points raised by referees in details. Their reports, together with Editorial comments, are below.

   If you are able to fully address these points, we would encourage you to submit a revised manuscript to Cellular & Molecular Biology Letters. Once you have made the necessary corrections, please submit online at:

   https://cmbl.editorialmanager.com/

   If you have forgotten your username or password please use the "Send Login Details" link to get your login information. For security reasons, your password will be reset.

   Please include a point-by-point response within the 'Response to Reviewers' box in the submission system and highlight (with 'tracked changes'/coloured/underlines/highlighted text) all changes made when revising the manuscript. Please ensure you describe additional experiments that were carried out and include a detailed rebuttal of any criticisms or requested revisions that you disagreed with. Please also ensure that your revised manuscript conforms to the journal style, which can be found in the Submission Guidelines on the journal homepage.

   The due date for submitting the revised version of your article is 30 Aug 2018.

   Please note, if your manuscript is accepted you will not be able to make any changes to the authors, or order of authors, of your manuscript once the editor has accepted your manuscript for publication. If you wish to make any changes to authorship before you resubmit your revisions, please reply to this email and ask for a 'Request for change in authorship' form which should be completed by all authors (including those to be removed) and returned to this email address. Please ensure that any changes in authorship fulfil the criteria for authorship as outlined in BioMed Central's editorial policies (http://www.biomedcentral.com/about/editorialpolicies#authorship).

   Once you have completed and returned the form, your request will be considered and you will be advised whether the requested changes will be allowed.
   By resubmitting your manuscript you confirm that all author details on the revised version are correct, that all authors have agreed to authorship and order of authorship for this manuscript and that all authors have the appropriate permissions and rights to the reported data.

   Please be aware that we may investigate, or ask your institute to investigate, any unauthorised attempts to change authorship or discrepancies in authorship between the submitted and revised versions of your manuscript.

   I look forward to receiving your revised manuscript soon.

   Best wishes,
   Aleksander F. Sikorski, Professor
   Cellular & Molecular Biology Letters
   https://cmbl.biomedcentral.com/

   Editorial remarks:

   1. The manuscript could become acceptable for publication only as Mini review. So please change the type of manuscript from Review to Mini review during resubmission.

   2. In Figs 5 and 6 please insert large A and B.

   3. Please remove "Fig. 6A" and "Fig. 6B" and type only "A" and "B". The legend should be: Common title. A...., B....

   4. In legends, where necessary, please add "With permission ...." and please add full reference.

"Upon re-submitting your revised manuscript, please upload your study's minimal underlying data set as either Supporting Information files or to a stable, public repository and include the relevant URLs, DOIs, or accession numbers within your revised cover letter. For a list of acceptable repositories, please see <http://journals.plos.org/plosone/s/data-availability#loc-recommended-repositories>. Any potentially identifying patient information must be fully anonymized. Important: If there are ethical or legal restrictions to sharing your data publicly, please explain these restrictions in detail. Please see our guidelines for more information on what we consider unacceptable restrictions to publicly sharing data: <http://journals.plos.org/plosone/s/data-availability#loc-unacceptable-data-access-restrictions>. Note that it is not acceptable for the authors to be the sole named individuals responsible for ensuring data access. We will update your Data Availability statement to reflect the information you provide in your cover letter.

(Response)

We have unified the title for consistency. Additionally, the file has been augmented with the relevant support information.

5. We notice that your supplementary table 1 are included in the manuscript file. Please remove them and upload them with the file type 'Supporting Information'. Please ensure that each Supporting Information file has a legend listed in the manuscript after the references list.

(Response)

We apologize for the oversight. Supplementary table 1 has been updated as Supporting Information accordingly.

6. Please review your reference list to ensure that it is complete and correct. If you have cited papers that have been retracted, please include the rationale for doing so in the manuscript text, or remove these references and replace them with relevant current references. Any changes to the reference list should be mentioned in the rebuttal letter that accompanies your revised manuscript. If you need to cite a retracted article, indicate the article's retracted status in the References list and also include a citation and full reference for theretraction notice.

(Response)

We have verified that the reference list is complete and correct.

**Reviewer #1:**

1. Figure 2B: both in the figure and in the text (line 241) the magnification and scale bars lack.

(Response)

Thank you for pointing this out. As such, the appropriate magnification levels and scale bars have been integrated into Figure 2B.

2. Line 287- line 291: P has to be connected with asterisks

(Response)

We apologize for this oversight. The missing asterisks have been inserted accordingly.

3. Please amplify the discussion pointing out the advantages of this

methods (PAT) compared to the others

(Response)

We appreciate your insightful recommendation. We have expanded our discussion to emphasize the benefits of the PAT method over alternative approaches. Under the new subheading “**Comparative efficacy of PAT grafting versus other treatments**” in section 4.8, we provide a comprehensive analysis, comparing PAT grafts with other clinically employed treatments for ischemic tissue-exposed wounds.

**Reviewer #2:**

There are some concerns:

1. I found a few grammatical errors.

(Response)

We have submitted the manuscript for an additional round of revision with Editage to ensure all grammatical errors are corrected.

2. Why perifascial areolar tissue (PAT) grafts is effective to promote granulation tissue formation that other tissues such as skin graft or pure fat doesn't.

(Response)

We appreciate your query. We have now incorporated a detailed comparison with skin grafting, as suggested. Current understanding acknowledges that full-thickness skin grafts have the potential to integrate with ischemic tissues. However, the specific mechanisms facilitating this process remain inadequately elucidated. Furthermore, the consensus is that such grafts are typically not indicated for application on ischemic tissues. We have reviewed in detail the reports on the methodology of skin grafting and incorporated them into our discussion, particularly emphasizing this in the “Vascular bridging” subheading in Section 4.2. It should be noted that our discussion does not encompass fat grafting, as this technique is predominantly applied to intact tissue rather than to open wounds.

3. Did you consider PAT viability after grafting on a bare area?

(Response)

We appreciate your invaluable suggestion. Our observations revealed that at 7 days post-application, vascular bridging was grossly visible in two of the six cases and 4 of 6 cases on histological analysis. By day 14, granulation tissue had nearly enveloped all exposed cartilage areas, obscuring the gross observation of vascular bridging, though vascular formation was present in all cases on tissue sections. Unlike skin grafts, the PAT grafts did not allow for a clear demarcation between viable and necrotic tissue on the dates of gross observation. Histological analysis also indicated areas of cellular attrition, yet a membranous structure persisted. Consequently, the precise determination of graft survival rates was challenging. Notably, granulation tissue formation was significantly accelerated by day 14, suggesting that even in the absence of vascular bridging, the grafts likely contributed to wound healing through their biomaterial properties, including stem content.

The aforementioned explanation has been incorporated into the manuscript in sections 3.3 “PAT graft survival,” 3.4.1 “Granulation tissue growth,” and 4.8.2 'Comparative Analysis with Other Treatment Modalities.”

4. What's the mechanism of action of PAT for granulation tissue formation, it's effect as a viable graft or it's effect when necrosis occurs and intra cellular mediators such as growth factors release? If first hypothesis is correct please mention what's the mechanism of PTA viability.

(Response)

We are grateful for your guidance. To address this, we have introduced a new subheading, 4.7 “Mechanisms underlying PAT graft viability.” This section is dedicated to elucidating the inferred biological mechanisms that contribute to the viability of PAT grafts.

5. You stated: "The open wound area, as determined from photographs, was significantly larger in the PAT 345 group than in the control group on day 7 (P < 0.05) (Fig 4A)". Does this mean wounds on both groups were not similar?.

(Response)

We apologize for the confusion and thank you for highlighting the ambiguity in this part. Wound healing principally involves two mechanisms: wound contraction and re-epithelialization. Given that wound contraction can lead to hypertrophic scarring, optimal treatment strategies aim to minimize this process, particularly in humans, without impeding overall wound closure. In our study, the PAT group exhibited a transient delay in wound closure due to reduced contraction by day 7, yet demonstrated an expedited re-epithelialization by day 14. Consequently, the total time to wound closure in the PAT group was found to be comparable to that of the control group by the 14^th^ day. This suggests that the observed process in the PAT group may represent an ideal paradigm for wound healing.

For easier understanding, we have added explanations in sections 3.4.3 “Wound contraction evaluation” and 4.6 “Wound contraction control with PAT graft.”

6. What's the mechanism of viability of PAT graft.

(Response)

We have added a section, 4.7, “Mechanisms underlying PAT graft viability,” accordingly. This new section provides a comprehensive summary of the currently understood biological processes contributing to the efficacy of PAT grafts.

7. You stated "The effectiveness of PAT grafts can be attributed to two primary factors, namely vascular bridging during graft survival and the provision of three essential elements that promote wound healing and angiogenesis: progenitor/stem cells, ECM molecules, and growth factors/cytokines.". My questions is why skin graft cannot do this? To best of our knowledge skin graft cannot cover or take on bare areas such as bone without periosteum but you concluded PAT can. Please clearly stat what's the difference.

(Response)

Thank you for highlighting the missing explanation. There has long been a theory that full-thickness skin grafts exhibit a “bridging” capability, enabling them to extend over ischemic tissue. However, as you correctly note, the current consensus now holds that such wounds are generally not amenable to grafting. After a detailed review of the literature, we have discussed the mechanism underlying this phenomenon, integrating our findings into section 4.2, “Vascular bridging.”

Furthermore, we have expanded on the distinctions and advantages of PAT grafting in comparison to traditional skin grafting. This comparative analysis, particularly focusing on the unique aspects of PAT grafting, is presented in section 4.8.1, “Distinctive aspects relative to skin grafting.”

8. I think the manuscript is too long and includes parts that are unnecessary.

(Response)

We've tried to cut down on unnecessary parts, but the manuscript has still become longer due to some added sentences. If you still think the manuscript includes parts that are unnecessary, could you please specify which parts you think are not needed?
