# Supplementary material for: Perifascial areolar tissue graft promotes angiogenesis and wound healing in an exposed ischemic component rabbit model
Source: PLoS One. 2024 Feb 20;19(2):e0298971. doi: 10.1371/journal.pone.0298971 (PMC10878522; doi:10.1371/journal.pone.0298971)
Supplement: Attachment — Submitted filename: renamed_1cfa0.docx [file pone.0298971.s004.docx]

**Response to the Reviewer’s comments**

Dear Editor;

Thank you for evaluating our manuscript and for your encouraging comments and appreciation of our work. Our manuscript has benefited from your comments and the reviewers’ guidance and thoughtful suggestions. We have read the comments provided by you and the reviewers and revised the manuscript accordingly. Please find our point-by-point responses to all the comments below.

**Reviewer #2:**

I read revised manuscript. The desired corrections have been made, but the writing style of the article is confusing for the reader. This is due to the lengthy and repetitive sentences, which make it tiresome to read and difficult to understand the main concept of the article.

(Response)

Thank you for pointing out this issue. We agree that the sentences were long and difficult to understand because of repetitive text. We modified the text to avoid repetition and improved understandability. Consequently, the word count has been reduced from 6340 to 5407 words. Thank you again.
